# Repeated dose (90 days) oral toxicity study of ursolic acid in Han-Wistar rats

**DOI:** 10.1016/j.toxrep.2020.04.005

**Published:** 2020-05-08

**Authors:** Lotte Geerlofs, Zhiyong He, Sa Xiao, Zhi-Cheng Xiao

**Affiliations:** a Department of Anatomy and Developmental Biology, Monash University, Melbourne, 3168, Australia; b iRiccorgpharm Health Pty Ltd, Melbourne, 3168, Australia

**Keywords:** Ursolic acid, Repeated dose toxicity, 90 days, No observed adverse effect level, OECD, A/G, Albumin/globulin ratio, ALB, Albumin, ALP, Alkaline phosphatase, ALT, Alanine aminotransferase, APTT, Activated partial thromboplastin time, AST, Aspartate aminotransferase, BASO, Basophils, CA, Calcium, CHOL, Cholesterol, CK, Creatine kinase, CL, Chloride, CREAT, Creatine, EOS, Eosinophils, FIB, Fibrogen, GGT, Gamma-glutamyl transferase, GLOB, Globulin, GLUC, Glucose, HGB, Haemoglobin, HPLC, High Performance Liquid Chromatography, HPMC, Hydroxypropyl methylcellulose, K, Potassium, LUC, Large unstained cells percent, LYMPH, Lymphocytes, MCH, Mean corpuscular haemoglobin, MCHC, Mean corpuscular haemoglobin concentration, MCV, Mean corpuscular volume, MONO, Monocytes percent, NA, Sodium, NEUT, Neutrophils percent, NOEL, No observed effect level, N.V.L, No visible lesions, OA, Oleanonic acid, OECD, Organisation for Economic Co-operation and Development, PHOS, Inorganic phosphate, PLT, Platelet count, PT, Prothrombin time, RBC, Red blood cell count, RDWG, Red blood cell distribution width gated, RETIC, Reticulocytes, TBIL, Total bilirubin, TPROT, Total protein, TRIG, Triglycerides, UA, Ursolic acid, WBC, White blood cell count

## Abstract

**Background:**

Ursolic acid (UA) has been used in alternative medicine for decades, and there has been a great interest in its medicinal properties. Despite this increased interest, a detailed long-term toxicity study has not been performed. The objective of this study was to determine the long-term toxic effect of UA on clinical chemistry, haematology, coagulation, pathology/morphology, behaviour and motor skills in rats.

**Methods:**

A solution was made by dissolving UA in a mixture of 0.1% Tween 80 and 0.5% hydroxypropyl methylcellulose in Milli-Q Water. The control group received the vehicle, and the test groups received a dose up to 1000 mg/kg/day via oral gavage. The solution was administered to both male and female (Han-Wistar) rats for 90 consecutive days.

**Results:**

UA did not cause any deaths, abnormal body weights or abnormal pathology at all test doses. In addition to that, no toxicological changes were observed in behaviour, neurotoxicity, coagulation, haematology or clinical chemistry that are related to the administration of UA.

**Conclusion:**

This study indicates that oral dosing of UA for 90 consecutive days does not lead to toxic effects at any of the doses. Therefore, the NOAEL for UA is likely to be higher than 1000 mg/kg/day.

## Highlights

• Ursolic acid was administered at a dose of 100, 300 or 1000 mg/kg/day.
• No Ursolic acid related effects were found after administrating orally for 90 days.
• The no observed adverse effect level is likely to be higher than 1000 mg/kg/day.

## Graphical abstract

**Figure:**
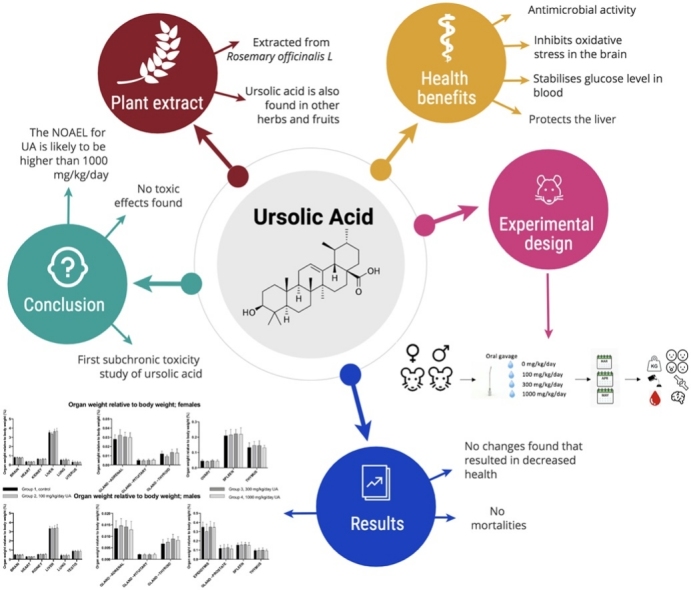

## 1 Introduction

Ursolic acid (UA) is a pentacyclic triterpenoid that can be isolated from different types of plant leaves and fruit waxes [1]. In the last two decades, many studies have been performed investigating the health benefits and the pharmacological relevance of UA and closely related compounds. UA has shown to possess multi-target properties and could serve as a therapeutic agent for several diseases. Studies have shown that UA inhibits excitotoxicity [2] and oxidative stress [3] in the brain and this could indicate that UA may have protective properties against several brain diseases. Furthermore, the possible effect of UA against liver diseases is well investigated, and UA protects the liver against various liver diseases like liver fibrosis [4] and fatty liver disease [5]. The loss of skeletal muscle might be inhibited by UA since it increases the skeletal muscle strength and synthesis of new tissue [5]. Additionally, UA decreases the pancreatic α-amylase activity and reduces the blood glucose level when investigating the effect of UA on diabetes [6,7]. Lastly, UA has shown antimicrobial properties against several gram-positive strains like *S.aureus* and *S.epidermidis* and gram-negative strains like *E.coli* [[8], [9], [10]].

For some of those diseases, the detailed mechanism has been researched. For example, UA increases the percentage of PPARγ positive cells in injured brain areas which could indicate that UA could maintain the metalloprotease/anti-metalloprotease balance in cerebral ischemia [11]. When used to treat liver conditions, UA decreases hepatic steatosis by activating PPAR-α (main regulator of hepatic lipid metabolism) in a non-alcoholic fatty liver model in rats [12,13]. Moreover, lastly, the administration of UA can hinder atherosclerosis-related parameters in a dose-dependent manner since it inhibits the proliferation of umbilical vein endothelial cells that is induced by C-reactive protein and interleukin 6 [14].

Besides being a therapeutic agent, Subramaniam et al. showed in 2015 that UA could serve as a food preservative since UA caused significant inhibition against food pathogens [15]. This was further confirmed when UA showed significant inhibition against mould and yeast strains [16]. All of these studies prove that UA is a well-investigated compound and could be applied in different fields.

Despite all those studies researching UA, the studies investigating the possible toxic effects of UA are almost non-existing. A study that combined UA and oleanolic acid (OA), which have similar pharmacological properties, showed that a single dose subcutaneous injection of 300 mg/kg did not result in any changes to the blood chemistry or organ morphology [17]. In addition to the single-dose study, a 5-day study was performed with OA at a dose of 1.0 mg/kg and the same administration route as the single-dose injection study. This experimental design did not lead to any mortalities but no necroscopy to check for changes in morphology was performed [18].

A proper long-term toxicity study of UA alone is currently non-existing. Therefore, this study was proposed. The objective of this study was to determine the possible toxic effects of UA at different doses. Rodents have been used to form a basis for identifying toxicities that are drug-exposed associated and therefore, a rat model is chosen to perform this study. To determine relevant and relative risks for other species, the most used biomarkers are haematology, body weights, clinical chemistry, organ weights, gross pathology changes and changes in physiologic functions [19]. For humans, the advised intake of UA supplements is between 150 and 300 mg per day. The possible toxic effects were investigated by administrating UA in different concentrations (100 mg/kg/day, 300 mg/kg/day and 1000 mg/kg/day) via oral gavage once a day for a total of 90 days to adult male and female rats and evaluating all the biomarkers mentioned above.

## 2 Material and methods

### 2.1 Ethics

The UK Home Office controls scientific procedures on animals in the UK and does so by the issue of licences under the Animals (Scientific Procedures) Act 1986. The regulations conform to EU Directive 2010/63/EU and achieve the standard of care required by the US Department of Health and Human Services' Guide for the Care and Use of Laboratory Animals. The project was approved by the Home Office under the PPL 70/8624, Toxicology of Chemicals, Protocol 2 ethics license.

### 2.2 Study design

The study design is presented in Table 1. The test and control items were administered to the appropriate animals by once-daily oral gavage from day 1 to 90. The volume for each animal was based on the most recent body weight measurement. The doses were given using a syringe with an attached gavage cannula and the first day of dosing was designated as day 1.

**Table 1:** Experimental design of the study with the doses, group size, and administered volumes.

Group No.	Treatment	Dose level (mg/kg/day)	Dose volume (ml/kg)	Dose concentration (mg/mL)	No. of animals	
					M	F
**1**	Control	0	10	0	10	10
**2**	UA	100	10	10	10	10
**3**	UA	300	10	30	10	10
**4**	UA	1000	10	100	10	10

### 2.3 Justification of route and preparation of doses

The gavage route of administration was selected for this study as this route is a possible route for human exposure. For this study, dose levels of 0, 100, 300, or 1000 mg/kg/day were selected based on the OECD (Organisation for Economic Co-operation and Development) testing guidelines that state that the limit for rodents is 1000 mg/kg/day when testing a chemical compound. The limit of 1000 mg/kg/day was chosen to cover all possible future doses that might want to be used in humans. The purity of UA was established by using High Performance Liquid Chromatography (HPLC) and was performed by Jiaherb Phytochem. The purity of UA was 92%, and the botanical source was *Rosmarinus officinalis* L. The control item, 0.5% hydroxypropyl methylcellulose (HPMC) (E4M), 0.1% Tween 80 in Milli-Q Water, was dispensed daily for administration to Group 1 control animals. The required amount of UA was weighed either into a weigh boat or directly into a mortar. 70% of the vehicle was weighed out and set aside. Small portions of the vehicle were mixed with UA using a pestle to obtain a homogeneous formulation. This was continued until all of the UA was incorporated into the formulation, and the mortar and pestle were then rinsed with the required amount of the 70% set aside vehicle. The formulation was then made to the final weight and mixed by continuous magnetic stirring and/or high shear mixing until the formulation was visibly homogeneous. The dosing formulations were prepared weekly, stored in a refrigerator set to maintain 4 °C, and dispensed daily. The dosing formulations were removed from the refrigerator and stirred for at least 30 min before dosing.

### 2.4 Animals

For this study, 40 male and 40 female Han-Wistar Crl:Han (WI) rats were received from Charles River UK Limited, Margate, Kent, UK. At the initiation of dosing, the animals were 6 to 7 weeks old. Each animal was identified using a subcutaneously implanted electronic cylindrical, ‘glass-sealed’ microchip. The animals were allowed to acclimate to the test facility rodent toxicology accommodation for a period of two weeks before the commencement of dosing. The animals were assigned to groups by a stratified randomisation scheme designed to achieve similar group mean body weights. Males and females were randomised separately. Animals were housed up to 5 per cage by sex, and all had environmental enrichments. They were housed in a 12-h light/12-h dark cycle except when interrupted for designated procedures.

The animals were fed a commercial diet in an expended form suitable for long term maintenance (Special Diet Services), and was provided ad libitum throughout the study, except during designated procedures and water was also provided ad libitum. Animals were observed twice daily, once at the start and once towards the end of the working day throughout the study for general health/mortality and moribundity.

## 3 Clinical observations

Animals were subjected to detailed clinical observations weekly from week -1 throughout the dosing period. They were observed regularly throughout the day on each day of dosing for signs of reaction to treatment, with particular attention being paid to the animals during and for the first hour after dosing. The body weight was measured daily from day 1 of the study. In addition to that, food consumption was measured weekly.

## 4 Ophthalmic examination

All animals were subject to ophthalmic examinations once during pretreatment. All control and high dose animals were also examined once during week 13. The eyes were examined using an indirect ophthalmoscope after the application of a mydriatic agent (1% Tropicamide, Mydriacyl®).

### 4.1 Detailed functional observations

Detailed functional observations were conducted once during pre-treatment and once during week 12 for all animals. These examinations were conducted by a technician not involved in the dosing procedures or in the collection of body weight and food consumption data. Before the independent technician entered the animal room to perform the examinations, the cage card showing treatment group was removed from each cage, leaving the second pre-prepared card as the functional observation animal identifier. In each cage, all animals had their tail marked with their functional observation battery number to allow the independent technician to identify each animal.

### 4.2 Home cage observations

Each animal was checked for prostration, stereotype/ bizarre behaviour, tremors, convulsions and ease of removal from the cage. In addition to that, rectal temperature was recorded in degrees Celsius.

### 4.3 Handling observations

The eyes were checked for pupillary function, miosis/ mydriasis, enophthalmos/exophthalmos, lacrimation and an evaluation of the diameter of the pupil was performed. Furthermore, the body tone, pinna response, the presence of salivation, overall ease of handling and the respiration rate and pattern were checked.

### 4.4 Air righting

Holding the animal in a supine position, it was dropped from approximately 30 cm, and the air righting response was rated.

### 4.5 Extensor thrust

The animal was grasped at the thorax gently from behind and raised off the surface in a vertical position. With their free hand, the observer gently but briskly pressed the tips of two fingers (or one finger and thumb) into the middle of the plantar surface (i.e. footpads) of each hind limb (one digit into each footpad). As the rodent extended the hind limbs, the presence/strength of the extensor thrust reflex was evaluated via digital palpation.

### 4.6 Observations in a standardised arena (2 min observation period)

The following parameters were observed: rearing, grooming, urination and defecation, arousal (level of alertness), posture, tremor (head, limbs, whole-body), convulsions, piloerection, palpebral closure, gait abnormalities and stereotypy (excessive repetition of behaviours) and/or unusual behaviours.

### 4.7 Functional tests

All animals had the following functional tests performed once during pre-treatment and once during the dosing period (Week 12). These assessments were performed at an approximately standardised time of day.

### 4.8 Grip strength

This was measured using a Dual/Single Channel Grip Strength Meter (Linton Instruments) to which wire screen assembly was attached. Once the animal gripped the screen, the body was pulled until its grasp was broken; the strain gauge records the force required. The procedure was repeated three times for the forelimbs and three times for the hindlimbs, and the mean fore and hind grip strengths calculated.

### 4.9 Pain perception

This was assessed by measurement of the tail-flick response, using a technique based on the method devised by D’Armour and Smith [20]. The apparatus used shone a calibrated infra-red heat source onto the tail and automatically measured the reaction time of the animal (accurate to 0.1 s). It was ensured that no visible injury to the tail was caused in this test.

### 4.10 Landing foot splay

Tempera paint was applied to the hind feet of each animal. The animal was then held in a horizontal, prone position with the nose approximately 30 cm above a bench surface covered with absorbent paper. When the animal was calm, it was dropped. The distance between the prints of the central footpads was measured, and the average measurement recorded. The procedure was repeated twice. If the rat did not land properly on its feet, this was recorded.

### 4.11 Motor activity

Each animal was placed in an individual cage held within a Smartframe utilising infra-red pyroelectric detectors. The movement was detected in 2 dimensions anywhere in the cage and was differentiated into basic and fine movements, and X and Y ambulation. Each animal was monitored for one session of 1 h, and activity counts being recorded over a successive period of 5 min each.

### 4.12 Sound and touch reaction

The reaction to a sudden sound, a click above the head, was recorded. Additionally, the reaction to touch on the rump with a blunt probe was recorded.

### 4.13 Clinical pathology

#### 4.13.1 Sample collection

In week 13, blood was collected from the jugular vein using sterile needles and disposable syringes. Urine was collected from animals housed in individual metabolism cages, with access to water only, over a period of approximately 6 h.

#### 4.13.2 Haematology

Blood samples were collected, transferred into tubes containing K_2_EDTA and analysed for the following parameters: red blood cell count, haemoglobin concentration, haematocrit, mean corpuscular volume, red blood cell distribution width, mean corpuscular haemoglobin concentration, mean corpuscular haemoglobin, white cell count and platelet count. In addition to that, the absolute values of the following parameters were measured: reticulocyte count, neutrophil count, lymphocyte count, monocyte count, eosinophil count, basophil count and large unstained cells. A blood smear was prepared from each haematology sample.

#### 4.13.3 Coagulation

Blood samples (0.5 mL) were collected, transferred into tubes containing 3.8% (w/v) trisodium citrate and processed for plasma and was analysed for the following parameters: activated partial thromboplastin time, fibrinogen, prothrombin time and sample quality.

#### 4.13.4 Clinical chemistry

Blood samples (0.7 mL) were collected, transferred into tubes containing lithium, heparin and processed for plasma. The following parameters were analysed: Alanine aminotransferase, aspartate aminotransferase, alkaline phosphatase, gamma-glutamyltransferase, creatine kinase, total bilirubin, urea, creatine, calcium, phosphate and total protein. In addition to that, albumin, globulin, albumin, glucose, cholesterol, triglycerides, sodium, potassium, chloride and the sample quality were analysed.

#### 4.13.5 Urinalyses

Urine samples were analysed for the following parameters: colour, appearance, specific gravity, volume, pH, protein, glucose, bilirubin, ketones and blood.

### 4.14 Euthanasia

Animals surviving until scheduled euthanasia were euthanized by a rising concentration of carbon dioxide, weighed and exsanguinated.

### 4.15 Necroscopy

All animals were subjected to a complete necroscopy examination, which included evaluation of the carcass and musculoskeletal system; all external surfaces and orifices; cranial cavity and external surfaced of the brain; and thoracic, abdominal and pelvic cavities with their associated organs and tissues.

#### 4.15.1 Organ weights

The following organs were weighed at necropsy for all animals (depending on the sex): brain, epididymis, adrenal gland, pituitary gland, prostate gland, thyroid gland, heart, kidney, liver, lung, ovary, spleen, testis, thymus and uterus. The epididymis, adrenal gland, kidney, ovary, and the testis are paired organs, and the duplicates of each of those organs were weighed together. The thyroid gland was weighed after fixation.

#### 4.15.2 Tissue preservation

Representative samples of the tissues presented in Table 2 were collected from all animals and preserved in 10% neutral buffered formalin except the bone marrow smear (air-dried, fixed in methanol), eye and optic nerve (preserved in Davidson’s fixative) and the testis (preserved in Modified Davidson’s fixative).

**Table 2:** Alphabetic list of collected tissue samples that were preserved and analysed later on.

Animal identification	Gland, mammary	Lesions/masses	Small intestine, ileum
Artery, aorta	Gland, parathyroid	Liver	Small intestine, jejunum
Bone marrow smear	Gland, pituitary	Lung	Spinal cord
Bone marrow, femur	Gland, prostate	Lymph node, mandibular	Spleen
Bone marrow, sternum	Gland, salivary, mandibular	Lymph node, mesenteric	Stomach
Bone, femur	Gland, seminal vesicle	Muscle, skeletal	Testis
Bone, sternum	Gland, thyroid	Nerve, optic	Thymus
Brain	Gut associated lymphoid tissue (Peyer’s patch)	Nerve, sciatic	Tongue
Cervix	Heart	Oesophagus	Trachea
Epididymis	Kidney	Ovary	Ureter
Eye	Large intestine, caecum	Oviduct	Urinary bladder
Gland, adrenal	Large intestine, colon	Pancreas	Uterus
Gland, harderian	Large intestine, rectum	Skin	Vagina
Gland, lacrimal	Larnyx	Small intestine, duodenum	

#### 4.15.3 Histology

Tissues identified in Table 2 (except animal identification and bone marrow smears) were embedded in paraffin, sectioned, mounted on glass slides and stained with haematoxylin and eosin.

### 4.16 Statistical analyses

Several analyses have been used to analyse all the data: one-way ANOVA, two way ANOVA and unpaired T-test. All statistical analyses were conducted at the 5% significance level. Numerical data collected on scheduled occasions for body weight, body weight gains, food consumption, haematology variables, coagulation variables, clinical chemistry variables, urinalysis variables, organ weights, organ weights relative to body weight, FOB quantitative variables and motor activity total response were analysed as indicated according to sex and occasion. All graphs were conducted with Graphpad prism version 8.

## 5 Results

### 5.1 Clinical observations

No premature deaths were recorded during this study; therefore, all the data from each animal was included in all analysis unless indicated otherwise.

#### 5.1.1 Body weight and food consumption

**Fig. 1:**
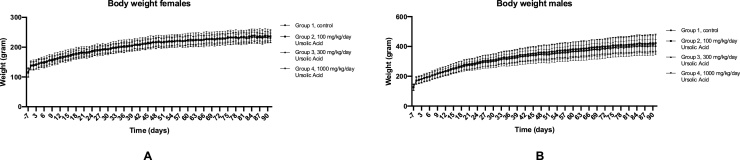
Average body weight of males and females throughout the experiment. A: Measured body weight of females on a daily basis. The error bars indicate ± SD. B: Measured body weight of males on a daily basis. The error bars indicate ± SD. No significant differences were found between the test groups and the control group in both sexes.

**Fig. 2:**
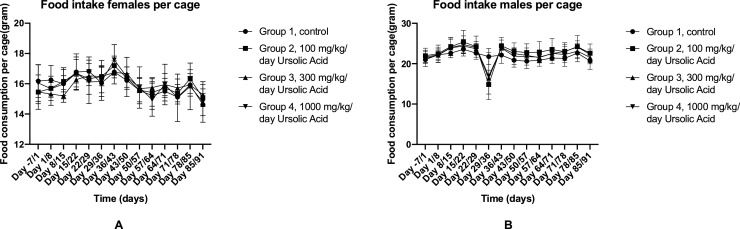
The foot intake of females and males throughout the experiment. A: The average food intake per cage of females. The food intake was measured every seven days. The error bars indicate SD. B: The average food intake per cage of males. The food intake was measured every seven days. The error bars indicate ± SD.

The body weight of the rats was measured on a daily basis. Statistical analysis showed that there was no difference found between the body weight of the experimental groups for both females (Fig. 1A) and males (Fig. 1B). Furthermore, there was no difference found between daily weight gain in both genders (data not shown). In addition to that, the food intake per cage was measured on a weekly basis. The food was provided ad libitum during the experiment. There was no difference between the food intake per cage for both females (Fig. 2A) and males (Fig. 2B), and the food intake of all groups was consistent during the full 90 days. However, there was a decrease in food intake in all test groups during day 29/36 timeframe; only the control group was consistent with their food intake in that particular time frame.

#### 5.1.2 Detailed clinical observations

There were no treatment-related intergroup differences in the functional observation battery parameters following UA administration at dose levels up to 1000 mg/kg/day. All of the behaviours exhibited and observations were considered to be typical for rats of this age and strain on this type of study conducted at the animal facility. The behaviour was observed on an observational battery, in the home cage, during handling and on a surface. There were no treatment-related ophthalmic findings in males or females that received UA at the dose levels up to 1000 mg/kg/day. Ophthalmic findings such as diffuse corneal, opacities or persistent pupillary membrane were observed in animals across all dose groups, including controls. These are considered to be typical findings observed at this laboratory in animals of this age and strain, in this type of study and are considered not to be related to the treatment.

#### 5.1.3 Quantitative function observations

There were no treatment-related inter-group differences in the quantitative functional observation parameters following UA administration at dose levels up to 1000 mg/kg/day (data presented in Table 3, Table 4, Table 5, Table 6). All values were greater than pre-treatment values which would be expected due to the age and the size of the animals. No statistical differences between the groups were found in the overall hind limb strength, forelimb grip strength, mean landing splay, and/or in-life locomotor observations that would indicate an effect on movement.

**Table 3:** Quantitative functional observational battery pretreatment observations of males. Mean + SD are given. * indicates significance (P = 0.05).

	Group 1, control	Group 2, 100 mg/kg/day UA	Group 3, 300 mg/kg/day UA	Group 4. 1000 mg/kg/day UA
**Arena rearing**	11.5 ± 2.5	9.9 ± 3.8	10.3 ± 3.4	8.2 ± 4.6
**Forelimb Grip strength mean (g)**	165.00 ± 26.50	167.40 ± 38.49	159.43 ± 37.66	172.46 ± 52.94
**Handlimb Grip strength mean (g)**	130.25 ± 19.55	132.14 ± 28.91	137.30 ± 19.91	130.09 ± 38.53
**Handlimb splay mean (cm)**	6.94 ± 0.46	8.18 ± 1.54	7.50 ± 1.66	7.40 ± 1.15
**Body temp (Celsius)**	37.40 ± 0.46	37.51 ± 0.43	37.41 ± 0.43	37.26 ± 0.25
**Tail flick (sec)**	2.170 ± 0.846	2.370 ± 0.469	2.110 ± 0.247	2.440 ± 0.369

**Table 4:** Quantitative functional observational battery in week 12 observations of males. Mean + SD are given. * indicates significance (P = 0.05).

	Group 1, control	Group 2, 100 mg/kg/day UA	Group 3, 300 mg/kg/day UA	Group 4. 1000 mg/kg/day UA
**Arena rearing**	4.9 ± 2.3	3.8 ± 2.8	4.9 ± 3.1	5.0 ± 3.0
**Forelimb Grip strength mean (g)**	296.4 ± 55.86	299.25 ± 71.28	249.76 ± 102.44	267.04 ± 78.26
**Handlimb Grip strength mean (g)**	228.36 ± 34.12	215.96 ± 33.28	219.91 ± 45.85	211.83 ± 49.98
**Handlimb splay mean (cm)**	11.22 ± 2.11	11.36 ± 3.00	12.16 ± 2.01	13.14 ± 1.48
**Body temp (Celsius)**	37.24 ± 0.71	37.18 ± 0.96	37.66 ± 0.79	37.37 ± 0.97
**Tail flick (sec)**	4.700 ± 1.332	4.350 ± 1.677	5.590 ± 1.581	5.670 ± 0.959

**Table 5:** Quantitative functional observational battery pretreatment observations of females. Mean + SD are given. * indicates significance (P = 0.05).

	Group 1, control	Group 2, 100 mg/kg/day UA	Group 3, 300 mg/kg/day UA	Group 4. 1000 mg/kg/day UA
**Arena rearing**	9.7 ± 4.8	11.5 ± 5.8	12.0 ± 3.3	12.5 ± 5.7
**Forelimb Grip strength mean (g)**	193.00 ± 46.98	152.89 ± 31.69	218.66 ± 170.80	168.46 ± 32.63
**Handlimb Grip strength mean (g)**	138.61 ± 45.04	126.74 ± 23.75	116.67 ± 32.72	135.55 ± 28.18
**Handlimb splay mean (cm)**	6.77 ± 0.90*	7.05 ± 1.07	6.21 ± 0.69	7.94 ± 1.03*
**Body temp (Celsius)**	67.70 ± 0.50	37.70 ± 0.58	37.56 ± 0.50	37.51 ± 0.52
**Tail flick (sec)**	2.929 ± 0.377	2.360 ± 0.918	3.257 ± 0.675	2.680 ± 0.819

**Table 6:** Quantitative functional observational battery in week 12 observations of females. Mean + SD are given. * indicates significance (P = 0.05).

	Group 1, control	Group 2, 100 mg/kg/day UA	Group 3, 300 mg/kg/day UA	Group 4. 1000 mg/kg/day UA
**Arena rearing**	6.4 ± 4.1	7.9 ± 5.9	6.3 ± 4.5	10.9 ± 6.4
**Forelimb Grip strength mean (g)**	284.70 ± 52.06	280.46 ± 61.68	307.76 ± 74.32	271.41 ± 78.96
**Handlimb Grip strength mean (g)**	230.47 ± 38.72	193.29 ± 46.60	205.03 ± 34.77	176.61 ± 53.94
**Handlimb splay mean (cm)**	9.71 ± 1.73	10.21 ± 1.17	9.66 ± 1.48	11.02 ± 1.85
**Body temp (Celsius)**	37.93 ± 0.85	38.16 ± 0.62	38.16 ± 0.90	38.34 ± 0.68
**Tail flick (sec)**	4.730 ± 1.786	4.910 ± 0.913	5.067 ± 0.752	4.320 ± 1.722

#### 5.1.4 Motor activity

There was no treatment-related inter-group difference in motor activity following UA administration of the different doses (data presented in Table 7). During the week 12 examination, it was noted that basic and fine movements were lower when compared to control values for males that received the low dose of 100 mg/kg/day between 41 and 50 min and for females that received the medium dose of 300 mg/kg/day between 26 and 30 min. However, these were isolated incidences, and there were no clinical observations notes, such as abnormal gait, increased/decreased activity or subdues behaviour that would suggest an effect on locomotor ability. Additionally, there was no relation to the dose. Therefore, these findings were considered to be incidental to the administration of UA.

**Table 7:** Summary of neurotoxicity screening: motor activity (movements/ 5 min): basic movements, fine movements, X-ambulation and Y-ambulation. The average of total movements + SD are given and * indicates significance (P = 0.05).

			Group 1, control	Group 2, 100 mg/kg/day UA	Group 3, 300 mg/kg/day UA	Group 4. 1000 mg/kg/day UA
**Basic** **movements**	**Male**	**Pretrial total**	2746.2 ± 538.1	2256.8 ± 723.5	2910.1 ± 634.2	2777.4 ± 800.0
		**Week 12 total**	3080 ± 955.4*	2678.2 ± 709.2	2556.6 ± 497.6	2290.9 ± 708.4*
	**Female**	**Pretrial total**	3548.1 ± 1367.4*	3252.6 ± 2015.9	3225.9 ± 977.4	2675.3 ± 929.5*
		**Week 12 total**	4374.5 ± 1975*	3093 ± 916.1*	3185.5 ± 858.5*	3556.4 ± 955.8*
**Fine** **movements**	**Male**	**Pretrial total**	2112.7 ± 447.1	1700.3 ± 566.6	2204.8 ± 543.1	2094.0 ± 607.8
		**Week 12 total**	2470.7 ± 716.2	2145.4 ± 582.2	2077.2 ± 425.2	1880.1 ± 582.5
	**Female**	**Pretrial total**	2662.7 ± 917.0	2604.0 ± 1459.0	2384.9 ± 658.9	2046 ± 668.0
		**Week 12 total**	3397.3 ± 1543.9*	2377.0 ± 761.0*	2431.7 ± 677.1*	2701.3 ± 751.3
**X-** **Ambulation**	**Male**	**Pretrial total**	228.9 ± 47.4	195.9 ± 76.0	258.1 ± 55.9	247.8 ± 81.1
		**Week 12 total**	181.3 ± 76.5*	149.7 ± 32.0	137.5 ± 43.1	109.4 ± 37.9*
	**Female**	**Pretrial total**	324.5 ± 163.1	345.2 ± 229.2	313.8 ± 129.6	230.3 ± 107.0
		**Week 12 total**	319.0 ± 139.2	225.6 ± 50.1	240.4 ± 58.0	280.9 ± 91.7
**Y-** **Ambulation**	**Male**	**Pretrial total**	407.2 ± 100.2	361.6 ± 127.8	452.4 ± 405.9	437.5 ± 156.5
		**Week 12 total**	450.9 ± 192.9	404.5 ± 125.6	360.0 ± 79.3	315.0 ± 130.8
	**Female**	**Pretrial total**	579.4 ± 334.5	595.6 ± 384.2	571.3 ± 233.8	407.4 ± 208.4
		**Week 12 total**	701.5 ± 331.0	508.5 ± 142.3	537.5 ± 163.1	605.0 ± 155.9

### 5.2 Necroscopy

#### 5.2.1 Organ weight

**Fig. 3:**
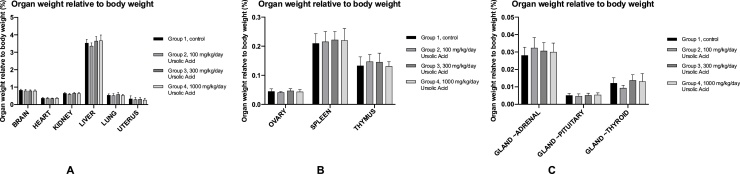
The organ weight relative to body weight of females. A: The organ weight relative to body weight of the brain, heart, kidney, liver, lung and uterus in. The average weight in grams ± SD is given. B: The organ weight relative to body weight of the ovary, spleen and thymus. The average weight in grams ± SD is given. C: The organ weight relative to body weight of the adrenal gland, pituitary gland and thyroid gland. The average weight in grams ± SD is given.

**Fig. 4:**
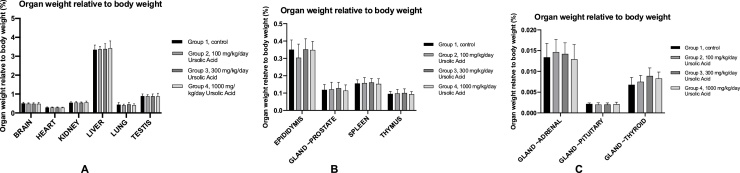
The organ weight relative to the body weigh in males. A: The organ weight relative to body weight of the brain, heart, kidney, liver, lung and testis. The average weight in grams ± SD is given. B: The organ weight relative to body weight of the epididymis, prostate gland, spleen and thymus. The average weight in grams ± SD is given. C: The organ weight relative to body weight of the adrenal gland, pituitary gland and thyroid gland. The average weight in grams ± SD is given.

All the organs that were weighed for all the female animals are presented in Fig. 3 A, B and C. To give a more accurate result, the actual weight is not used for these graphs. The graphs represent the percentage of that organ weight relative to the body weight. Statistical analysis was performed on the data sets, and it showed that all experimental groups had similar organ weights relative to the body weight than the control group. The only difference in weighed organs between males and females were their reproductive organs. The organ weight relative to the body weight of males are presented in Fig. 4 A, B and C. All of the percentile organ weights were similar across the experimental groups and the control group. Therefore, daily oral administration of UA at a dose up to 1000 mg/kg/day does not have an effect on the organ weight in females and males.

#### 5.2.2 Gross pathology and histopathology

**Fig. 5:**
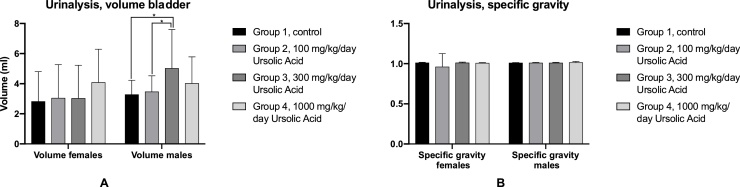
Urinalysis results. A: Average volume for females and males. Error bars indicate SD and * indicates significance (<0.05). There was no difference found in females but in males, the medium dose had a significant higher volume than the control and low dose group. B: Average specific gravity in females and males. No significant differences were found between groups. Error bars indicate SD.

For the gross pathology, several tissue samples were macroscopically observed for visible lesions. For each tissue sample, depending on the sex of the animal, all animals were investigated unless indicated otherwise. The gross pathology findings for both males and females are presented in Table 8, Table 9, Table 10, Table 11, Table 12, Table 13 and are presented in alphabetic order. The majority of the organs were not affected at all in both the control and the test groups. The two organs that seemed to be affected in both the test groups and the control groups were the lungs and thymus. The lungs of the male subjects were affected more often than the lungs of the female subjects and the discolouration varied per group. The observations of the thymus were similar to the lungs across both sexes and groups. Lesions were found in both the control and test groups, and the only group that did not have visible lesions was the low dose in females. The prevalence of the visible lesions was similar across the test and control group for both males and females. Therefore, this could indicate that these lesions are not related to the administration of UA. Since the gross pathology showed that no statistical differences for all parameters between groups were detected, the histopathology (Supplementary data) mainly focussed on the comparison of the control group and the high dose of 1000 mg/kg/day. This decision was made since any possible difference of lesions would be most likely found in the high dose group. The low dose (100 mg/kg/day) and medium-dose (300 mg/kg/day) were only included in the histopathology for the lungs and thymus since gross pathology showed that lesions were found across all groups for those two organs. The number of sections for those two organs for the low and medium dose were lower than 10. For the control group and the high dose group, the number of sections per group was 10 unless the investigated tissue was not present in that section. Histopathology showed similar results than the gross pathology. The majority of the tissues did not show any lesions in the high dose group or the control group. Possible lesions in the control group are ‘natural’ lesions since these animals did not receive any UA. Since the occurrence of the lesions in both the lungs and thymus were similar between the test groups and the control group, this would even further indicate that these lesions are not related to the administration of UA at different doses (Fig. 5).

**Table 8:** Summary of the gross pathology findings; effect of UA on the aorta, bone structures, brain, cervix, epididymis, esophagus and eye. N.V.L = no visible lesions. No lesions were found in these tissues.

		Male				Female			
		Group 1, Control	Group 2, 100 mg/kg/day	Group 3, 300 mg/kg/day	Group 4, 1000 mg/kg/day	Group 1, Control	Group 2, 100 mg/kg/day	Group 3, 300 mg/kg/day	Group 4, 1000 mg/kg/day
	**Number of animals**	10	10	10	10	10	10	10	10
**Artery, aorta**	Submitted	10	10	10	10	10	10	10	10
	N.V.L	10	10	10	10	10	10	10	10
**Bone, femur**	Submitted	10	10	10	10	10	10	10	10
	N.V.L	10	10	10	10	10	10	10	10
**Bone, sternum**	Submitted	10	10	10	10	10	10	10	10
	N.V.L	10	10	10	10	10	10	10	10
**Bone marrow, femur**	Submitted	10	10	10	10	10	10	10	10
	N.V.L	10	10	10	10	10	10	10	10
**Bone marrow, sternum**	Examined	10	10	10	10	10	10	10	10
	N.V.L	10	10	10	10	10	10	10	10
**Bone marrow, smear**	Submitted	10	10	10	10	10	10	10	10
	N.V.L	10	10	10	10	10	10	10	10
**Brain**	Submitted	10	10	10	10	10	10	10	10
	N.V.L	10	10	10	10	10	10	10	10
**Cervix**	Submitted	–	–	–	–	10	10	10	10
	N.V.L	–	–	–	–	10	10	10	10
**Epididymis**	Submitted	10	10	10	10	–	–	–	–
	N.V.L	10	10	10	10	–	–	–	–
**Esophagus**	Submitted	10	10	10	10	10	10	10	10
	N.V.L	10	10	10	10	10	10	10	10
**Eye**	Submitted	10	10	10	10	10	10	10	10
	N.V.L	10	10	10	10	10	10	10	10

**Table 9:** Summary of the gross pathology findings; effect of UA on the galt and glands N.V.L = no visible lesions. No lesions were found in these tissues in the test groups.

		Male				Female			
		Group 1, Control	Group 2, 100 mg/kg/day	Group 3, 300 mg/kg/day	Group 4, 1000 mg/kg/day	Group 1, Control	Group 2, 100 mg/kg/day	Group 3, 300 mg/kg/day	Group 4, 1000 mg/kg/day
	**Number of animals**	10	10	10	10	10	10	10	10
**Galt**	Submitted	10	10	10	10	10	10	10	10
	N.V.L	10	10	10	10	10	10	10	10
**Gland, adrenal**	Submitted	10	10	10	10	10	10	10	10
	N.V.L	10	10	10	10	10	10	10	10
**Gland, harderian**	Submitted	10	10	10	10	10	10	10	10
	N.V.L	10	10	10	10	10	10	10	10
**Gland, lacrimal**	Submitted	10	10	10	10	10	10	10	10
	N.V.L	10	10	10	10	10	10	10	10
**Gland, mammary**	Examined	10	10	10	10	10	10	10	10
	N.V.L	10	10	10	10	10	10	10	10
**Gland, parathyroid**	Submitted	10	10	10	10	10	10	10	10
	N.V.L	10	10	10	10	10	10	10	10
**Gland, pituitary**	Submitted	10	10	10	10	9	10	10	10
	N.V.L	10	10	10	10	9	10	10	10
	Not examined; lost during necroscopty	–	–	–	–	1	0	0	0
**Gland, prostate**	Submitted	10	10	10	10	–	–	–	–
	N.V.L	10	10	10	10	–	–	–	–
**Gland, salivary. mandibular**	Submitted	10	10	10	10	10	10	10	10
	N.V.L	10	10	10	10	10	10	10	10
**Gland, seminal vesicle**	Submitted	10	10	10	10	–	–	–	–
	N.V.L	10	10	10	10	–	–	–	–

**Table 10:** Summary of the gross pathology findings; effect of UA on the thyroid gland, heart, kidney, large intestines, larynx and liver. N.V.L = no visible lesions. Lesions were only found on/in the liver in males that were administered a medium dose of 300 mg/kg/day.

		Male				Female			
		Group 1, Control	Group 2, 100 mg/kg/day	Group 3, 300 mg/kg/day	Group 4, 1000 mg/kg/day	Group 1, Control	Group 2, 100 mg/kg/day	Group 3, 300 mg/kg/day	Group 4, 1000 mg/kg/day
	**Number of animals**	10	10	10	10	10	10	10	10
**Gland, thyroid**	Submitted	10	10	10	10	10	10	10	10
	N.V.L	10	10	10	10	10	10	10	10
**Heart**	Submitted	10	10	10	10	10	10	10	10
	N.V.L	10	10	10	10	10	10	10	10
**Kidney**	Submitted	10	10	10	10	10	10	10	10
	N.V.L	10	10	10	10	10	10	10	10
**Large intestine, cecum**	Submitted	10	10	10	10	10	10	10	10
	N.V.L	10	10	10	10	10	10	10	10
**Large intestine, colon**	Examined	10	10	10	10	10	10	10	10
	N.V.L	10	10	10	10	10	10	10	10
**Large intestine, rectum**	Submitted	10	10	10	10	10	10	10	10
	N.V.L	10	10	10	10	10	10	10	10
**Larynx**	Submitted	10	10	10	10	10	10	10	10
	N.V.L	10	10	10	10	10	10	10	10
**Liver**	Submitted	10	10	10	10	10	10	10	10
	N.V.L	10	10	7	10	10	10	10	10
	Prominent lobular architecture	0	0	2	0	0	0	0	0
	Abnormal appearance	0	0	1	0	0	0	0	0

**Table 11:** Summary of the gross pathology findings; effect of UA on the lung, lymph node, muscle, nerves, ovary, oviduct and pancreas. N.V.L = no visible lesions. Lesions were found on/in the lung in both the test groups and the control group in both sexes.

		Male				Female			
		Group 1, Control	Group 2, 100 mg/kg/day	Group 3, 300 mg/kg/day	Group 4, 1000 mg/kg/day	Group 1, Control	Group 2, 100 mg/kg/day	Group 3, 300 mg/kg/day	Group 4, 1000 mg/kg/day
	**Number of animals**	10	10	10	10	10	10	10	10
**Lung**	Submitted	10	10	10	10	10	10	10	10
	N.V.L	4	5	5	5	7	9	9	9
	Focus	0	0	1	0	0	0	0	0
	Focus; dark	1	3	1	2	0	0	0	1
	Focus; pale	0	0	0	1	1	1	0	0
	Discoloration; mottled	4	3	3	2	2	0	1	0
**Lymph node, mesenteric**	Submitted	10	10	10	10	10	10	10	10
	N.V.L	10	10	10	10	10	10	10	10
**Muscle, skeletal**	Submitted	10	10	10	10	10	10	10	10
	N.V.L	10	10	10	10	10	10	10	10
	Infiltration, mononuclear cell, minimal	10	10	10	10	10	10	10	10
**Nerve, optic**	Submitted	10	10	10	10	10	10	10	10
	N.V.L	10	10	10	10	10	10	10	10
	Not examined, not present	10	10	10	10	10	10	10	10
**Nerve, sciatic**	Submitted	10	10	10	10	10	10	10	10
	N.V.L	10	10	10	10	10	10	10	10
**Ovary**	Examined	–	–	–	–	10	10	10	10
	N.V.L	–	–	–	–	10	10	10	10
**Oviduct**	Submitted	–	–	–	–	10	10	10	10
	N.V.L	–	–	–	–	10	10	10	10
**Pancreas**	Submitted	10	10	10	10	10	10	10	10
	N.V.L	10	10	10	10	10	10	10	10

**Table 12:** Summary of the gross pathology findings; effect of UA on the skin, small intestines, spinal cord, spleen, stomach, tail, testis and thymus. N.V.L = no visible lesions. Lesions were found in/on the thymus across all test groups and control groups in both males and females.

		Male				Female			
		Group 1, Control	Group 2, 100 mg/kg/day	Group 3, 300 mg/kg/day	Group 4, 1000 mg/kg/day	Group 1, Control	Group 2, 100 mg/kg/day	Group 3, 300 mg/kg/day	Group 4, 1000 mg/kg/day
	**Number of animals**	10	10	10	10	10	10	10	10
**Skin**	Submitted	10	10	10	10	10	10	10	10
	N.V.L	10	10	10	10	10	10	10	10
	Thin hair coat								
**Small intestine, duodenum**	Submitted	10	10	10	10	10	10	10	10
	N.V.L	10	10	10	10	10	10	10	10
**Small intestine, ileum**	Submitted	10	10	10	10	10	10	10	10
	N.V.L	10	10	10	10	10	10	10	10
**Small intestine, jejunum**	Submitted	10	10	10	10	10	10	10	10
	N.V.L	10	10	10	10	10	10	10	10
**Spinal cord**	Examined	10	10	10	10	10	10	10	10
	N.V.L	10	10	10	10	10	10	10	10
**Spleen**	Submitted	10	10	10	10	10	10	10	10
	N.V.L	10	10	10	10	10	10	10	10
**Stomach**	Submitted	10	10	10	10	10	10	10	10
	N.V.L	10	10	10	10	10	10	10	10
**Tail**	Submitted	–	–	–	–	0	0	1	0
	Scab	–	–	–	–	–	–	1	–
**Testis**	Submitted	0	0	2	0	–	–	–	–
	N.V.L	0	0	1	0	–	–	–	–
**Thymus**	Submitted	10	10	10	10	10	10	10	10
	N.V.L	5	7	7	5	6	10	8	7
	Focus	0	1	0	0	0	0	0	0
	Focus; dark	5	2	3	5	4	0	2	3

**Table 13:** Summary of the gross pathology findings; effect of UA on the tongue, trachea, ureter, urinary bladder, uterus and vagina. N.V.L = no visible lesions. Calculus was found in the urinary bladder in two animals, one in the low dose group and one in the medium dose group in males.

		Male				Female			
		Group 1, Control	Group 2, 100 mg/kg/day	Group 3, 300 mg/kg/day	Group 4, 1000 mg/kg/day	Group 1, Control	Group 2, 100 mg/kg/day	Group 3, 300 mg/kg/day	Group 4, 1000 mg/kg/day
	**Number of animals**	10	10	10	10	10	10	10	10
**Tongue**	Submitted	10	10	10	10	10	10	10	10
	N.V.L	10	10	10	10	10	10	10	10
**Trachea**	Submitted	10	10	10	10	10	10	10	10
	N.V.L	10	10	10	10	10	10	10	10
**Ureter**	Submitted	10	10	10	10	10	10	10	10
	N.V.L	10	10	10	10	10	10	10	10
**Urinary bladder**	Submitted	10	10	10	10	10	10	10	10
	N.V.L	10	9	9	10	10	10	10	10
	Calculus	0	1	1	0	0	0	0	0
**Uterus**	Examined	–	–	–	–	10	10	10	10
	N.V.L	–	–	–	–	10	10	10	10
**Vagina**	Submitted	–	–	–	–	10	10	10	10
	N.V.L	–	–	–	–	10	10	10	10

### 5.3 Haematology, coagulation and urinalysis

Haematology and coagulation analysis for all parameters described in the material and methods was performed after the 90 days. The average concentration of the different cells in males are presented in Table 14. For the majority of the parameters, there is no difference between the groups and UA has no effect on the concentration of the cells present in blood. However, the platelet count of the 300 mg/kg/day group (p=<0.0001) and 1000 mg/kg/day group (p = 0.0156) was significantly increased in comparison with the control group. In addition to that, the 300 mg/kg/day group also has an increased platelet count compared to the lower dose of 100 mg/kg/day (p= <0.0001) and the high dose group of 1000 mg/kg/day (p = 0.0062). The exact same parameters were used in the blood analysis in females, and those results are presented in Table 15. The concentrations of almost all of the cells were similar across the test and control group. Similar to the males, there were inconsistencies in the platelet count. The low dose group (100 mg/kg/day) had the lowest count of the four groups and was significantly lower than the control group (p= <0.0001), the medium (300 mg/kg/day) (p=<0.0001) and high dose (1000 mg/kg/day) (p = 0.0002). Compared to the males, that is different since the control group had the lowest concentration of platelets in the bloodstream and the medium dose the highest concentration.

**Table 14:** Haematology and coagulation results of males. The average + SD is given. *, ^A,B^ indicates significant difference, p < 0.05. A significant difference was found between the control group and the medium and high dose. The platelet count of those two groups were raised compared to the control group. In addition to that, the medium dose group had a significantly higher platelet count than the low dose and high dose group.

	Group 1, Control	Group 2, 100 mg/kg/day UA	Group 3, 300 mg/kg/day UA	Group 4, 1000 mg/kg/day UA
**APTT**	14.57 ± 0.873	15.66 ± 1.372	15.25 ± 0.525	15.42 ± 1.391
**BASO**	0.011 ± 0.004	0.010 ± 0	0.012 ± 0.004	0.013 ± 0.005
**EOS**	0.136 ± 0.070	0.120 ± 0.035	0.102 ± 0.040	0.129 ± 0.032
**FIB**	2.132 ± 0.149	2.056 ± 0.347	2.130 ± 0.163	2.225 ± 0.158
**HCT**	0.474 ± 0.031	0.482 ± 0.017	0.478 ± 0.200	0.491 ± 0.026
**HGB**	162.8 ± 6.923	164.1 ± 6.194	162.8 ± 3.584	165.3 ± 6.519
**LUC**	0.039 ± 0.027	0.041 ± 0.011	0.044 ± 0.016	0.043 ± 0.010
**LYMPH**	4.246 ± 0.938	4.778 ± 0.588	4.319 ± 0.969	4.211 ± 0.694
**MCH**	18.11 ± 0.494	17.69 ± 0.987	17.58 ± 0.991	17.42 ± 0.845
**MCHC**	344.0 ± 9.366	340.7 ± 9.734	340.6 ± 9.652	337.1 ± 8.580
**MCV**	52.68 ± 0.851	51.89 ± 1.668	51.57 ± 1.746	51.70 ± 2.061
**MONO**	0.086 ± 0.029	0.110 ± 0.041	0.097 ± 0.035	0.110 ± 0.034
**NEUT**	0.898 ± 0.191	0.938 ± 0.228	0.790 ± 0.247	1.024 ± 0.182
**PLT**	712.5 ± 91.06*	726.0 ± 115.5^A^	829.6 ± 76.56*^A,B^	770.0 ± 292.6*^,B^
**PT**	10.36 ± 0.260	10.38 ± 0.399	10.33 ± 0.241	10.28 ± 0.273
**RBC**	8.994 ± 0.543	9.294 ± 0.364	9.299 ± 0.638	9.819 ± 1.097
**RDWG**	12.24 ± 0.307	12.61 ± 0.411	12.80 ± 0.707	27.79 ± 45.18
**RETIC**	169.5 ± 15.69	163.9 ± 16.14	167.1 ± 19.66	154.9 ± 60.61
**WBC**	5.413 ± 1.170	5.990 ± 0.466	5.361 ± 1.074	5.609 ± 0.758

**Table 15:** Haematology and coagulation results of females. The average + SD is given. *, ^A,B^ indicates significant difference, p < 0.05. A significant difference was found between the low dose group and the control group, medium dose group and high dose group. The platelet count of the low dose group was decreased compared to the other groups.

	Group 1, Control	Group 2, 100 mg/kg/day UA	Group 3, 300 mg/kg/day UA	Group 4, 1000 mg/kg/day UA
**APTT**	14.06 ± 0.529	15.08 ± 3.887	12.86 ± 3.840	12.40 ± 3.380
**BASO**	0.010 ± 0	0.010 ± 0	0.010 ± 0	0.010 ± 0
**EOS**	0.083 ± 0.026	0.071 ± 0.024	0.094 ± 0.027	0.084 ± 0.013
**FIB**	1.672 ± 0.213	0.543 ± 0.317	1.614 ± 0.186	1.700 ± 0.140
**HCT**	0.457 ± 0.015	0.460 ± 0.025	0.446 ± 0.014	0.460 ± 0.029
**HGB**	158.3 ± 4.233	157.4 ± 7.518	155.0 ± 2.500	158.4 ± 5.659
**LUC**	0.026 ± 0.014	0.026 ± 0.016	0.024 ± 0.007	0.023 ± 0.010
**LYMPH**	2.969 ± 0.789	3.212 ± 0.909	2.977 ± 0.357	3.257 ± 0.613
**MCH**	18.92 ± 0.442	18.81 ± 0.635	18.78 ± 0.733	18.78 ± 0.806
**MCHC**	346.6 ± 6.433	342.6 ± 9.275	347.7 ± 9.274	345.1 ± 10.96
**MCV**	54.59 ± 1.487	54.94 ± 1.421	54.12 ± 1.704	54.42 ± 1.618
**MONO**	0.073 ± 0.024	0.087 ± 0.044	0.072 ± 0.020	0.072 ± 0.012
**NEUT**	0.728 ± 0.212	0.678 ± 0.114	0.701 ± 0.186	0.660 ± 0.232
**PLT**	805.8 ± 88.83*	749.7 ± 104.6*^,A,B^	805.7 ± 100.3^A^	800.6 ± 108.8^B^
**PT**	10.00 ± 0.226	10.28 ± 1.105	9.911 ± 0.232	9.882 ± 0.083
**RBC**	8.371 ± 0.248	8.378 ± 0.530	8.253 ± 0.318	5.452 ± 0.527
**RDWG**	10.69 ± 0.296	10.88 ± 0.415	10.71 ± 0.431	10.67 ± 0.418
**RETIC**	165.4 ± 30.20	180.8 ± 51.59	157.3 ± 45.60	159.4 ± 45.60
**WBC**	3.889 ± 0.849	4.100 ± 0.990	3.873 ± 0.402	4.099 ± 0.621

The volume and specific gravity were measured for part of the urinalysis. The volume for males and females are presented in Table 5A and the specific gravity in 5B. The specific gravity could indicate whether the kidneys were working properly. There were no inconsistencies found in the specific gravity or volume of the bladder in females. In males, the medium dose of 300 mg/kg/day had a significantly higher volume than the control group (p = 0.0099) and the low dose group of 100 mg/kg/day (p = 0.0265), but the specific gravity of the control group and test groups were similar to each other. Furthermore, the analysis of the clarity of the urine, colour of the urine, the pH, protein levels, glucose levels, bilirubin levels, ketones levels and blood levels showed that there was no significant difference found across all groups (data not presented).

### 5.4 Clinical chemistry

All of the clinical chemistry parameters and their concentration present in blood are given in Table 16 for males and Table 17 for females. The performed statistical analysis showed that there is no difference for most of the parameters in concentration for both males and females when comparing the control group with the test groups. There was a difference found in the concentration of creatine kinase (CK) in both males and females. In males, the control group had a higher concentration compared to the low dose group (p = 0.0080), medium-dose group (p = 0.0062) and high dose group (p=<0.0001).

**Table 16:** Clinical chemistry summary; the average concentration + SD of parameters in males is given. The CK concentration of the control group was significantly higher than all UA test groups. In addition to that, the low and medium dose had a higher concentration than the high dose group. * and ^A,B^ indicates significant difference, p < 0.05.

		Group 1, Control	Group 2, 100 mg/kg/day UA	Group 3, 300 mg/kg/day UA	Group 4, 1000 mg/kg/day UA
Parameter	Unit				
**AST**	units/L	62.5 ± 5.3	62.6 ± 6.2	62.0 ± 6.2	62.0 ± 6.8
**ALT**	units/L	36.1 ± 8.0	43.1 ± 7.0	37.8 ± 5.7	40.9 ± 11.8
**ALP**	units/L	109.1 ± 28.9	112.5 ± 26.2	121.7 ± 19.5	106.9 ± 22.4
**GGT**	units/L	2.0 ± 0.0	2.0 ± 0.0	2.0 ± 0.0	2.0 ± 0.0
**CK**	units/L	226.9 ± 136.2*	194.9 ± 87.7*^,B^	194.1 ± 80.3*^,A^	155.9 ± 67.5*^,A,B^
**TBIL**	mmol/L	1.30 ± 0.00	1.30 ± 0.00	1.30 ± 0.00	1.30 ± 0.00
**UREA**	mmol/L	5.76 ± 0.46	5.81 ± 0.57	5.51 ± 0.45	5.74 ± 0.30
**CREAT**	μmol/L	33.2 ± 4.0	31.2 ± 3.7	30.9 ± 5.1	31.9 ± 4.1
**GLUC**	mmol/L	6.363 ± 0.735	6.851 ± 0.922	5.852 ± 1.257	6.433 ± 0.759
**CHOL**	mmol/L	2.05 ± 0.30	1.87 ± 0.38	1.97 ± 0.31	1.86 ± 0.16
**TRIG**	gram/L	1.332 ± 0.547	1.651 ± 0.435	1.731 ± 0.812	1.394 ± 0.443
**TPROT**	gram/L	71.1 ± 1.5	70.1 ± 1.7	70.2 ± 2.0	71.3 ± 2.8
**ALB**	gram/L	45.0 ± 1.7	44.7 ± 1.8	44.9 ± 2.9	45.3 ± 1.3
**GLOB**	gram/L	26.4 ± 1.8	25.6 ± 2.0	25.3 ± 2.4	26.1 ± 2.1
**A/G**	n.a	1.72 ± 0.19	1.76 ± 0.20	1.80 ± 0.28	1.74 ± 0.12
**PHOS**	mmol/L	2.745 ± 0.063	2.474 ± 0.051	2.726 ± 0.047	2.761 ± 0.050
**CA**	mmol/L	1.988 ± 0.234	1.994 ± 0.202	1.814 ± 0.194	1.899 ± 0.119
**NA**	mmol/L	144.2 ± 1.9	144.5 ± 1.1	144.5 ± 1.2	144.5 ± 1.1
**K**	mmol/L	4.99 ± 0.33	5.04 ± 0.28	4.80 ± 0.26	5.08 ± 0.21
**CL**	mmol/L	101.2 ± 1.4	101.8 ± 1.0	102.3 ± 1.1	101.8 ± 1.1

**Table 17:** Clinical chemistry summary; the average concentration + SD of parameters in females is given. The control group and the low dose group had a higher CK concentration than the medium and high dose group. * and ^A,B^ indicates significant difference, p < 0.05.

		Group 1, Control	Group 2, 100 mg/kg/day UA	Group 3, 300 mg/kg/day UA	Group 4, 1000 mg/kg/day UA
Parameter	Unit				
**AST**	units/L	62.0 ± 4.8	70.0 ± 15.3	53.3 ± 5.9	58.7 ± 13.1
**ALT**	units/L	42.2 ± 6.7	51.5 ± 27.3	41.6 ± 11.1	44.7 ± 7.8
**ALP**	units/L	63.4 ± 20.1	63.4 ± 16.5	58.0 ± 11.0	68.6 ± 23.9
**GGT**	units/L	2.0 ± 0.0	2.0 ± 0.0	2.0 ± 0.00	2.0 ± 0.0
**CK**	units/L	162.5 ± 67.6*	156.7 ± 103.4^A^	100.1 ± 42.4*	102.2 ± 29.7^A^
**TBIL**	mmol/L	1.30 ± 0.00	1.30 ± 0.00	1.30 ± 0.00	1.30 ± 0.00
**UREA**	mmol/L	5.68 ± 0.71	5.88 ± 0.72	5.72 ± 0.79	5.85 ± 0.58
**CREAT**	μmol/L	36.8 ± 4.5	38.9 ± 4.7	33.3 ± 3.2	34.3 ± 10.0
**GLUC**	mmol/L	6.749 ± 0.647	6.141 ± 0.484	7.161 ± 1.011	6.392 ± 0.943
**CHOL**	mmol/L	1.75 ± 0.25	1.82 ± 0.39	1.63 ± 0.46	1.55 ± 0.41
**TRIG**	gram/L	1.450 ± 0.680	1.454 ± 0.702	1.360 ± 0.439	1.093 ± 0.541
**TPROT**	gram/L	74.0 ± 3.4	74.5 ± 4.7	70.2 ± 3.4	72.7 ± 5.5
**ALB**	gram/L	51.7 ± 3.0	51.8 ± 3.6	48.9 ± 2.4	50.3 ± 3.5
**GLOB**	gram/L	22.6 ± 2.3	22.6 ± 2.7	21.2 ± 2.4	22.6 ± 2.5
**A/G**	n.a	2.33 ± 0.33	2.31 ± 0.32	2.34 ± 0.31	2.34 ± 0.23
**PHOS**	mmol/L	2.803 ± 0.064	2.814 ± 0.112	2.737 ± 0.090	2.797 ± 0.077
**CA**	mmol/L	1.526 ± 0.435	1.658 ± 0.379	1.642 ± 0.246	1.666 ± 0.299
**NA**	mmol/L	144.9 ± 1.1	145.2 ± 2.6	143.4 ± 1.3	145.1 ± 2.0
**K**	mmol/L	5.24 ± 0.61	5.51 ± 0.66	5.00 ± 0.36	5.42 ± 0.65
**CL**	mmol/L	102.2 ± 1.9	103.0 ± 2.3	101.4 ± 1.4	102.4 ± 0.8

Furthermore, there was also a difference found between the low dose group and the high group (p = 0.0006) and the medium-dose group and the high dose group (p = 0.0009). In females, the control group had a significantly higher concentration than the medium-dose group (p=<0.0001) and the high dose group (p=<0.0001). The low dose concentration also had a significantly higher CK concentration than the medium-dose group (p = 0.0001) and the high dose group (p = 0.0001).

## 6 Discussion

There has been an increasing interest in UA as a potential medicine. In 2010, 150 articles were published investigating the medicinal properties of UA, but in 2015 the number of published articles researching UA almost doubled [21]. UA has been used in alternative medicine for decades, and researchers have concluded that UA could serve as a medicinal agent. For instance, UA has shown to have neuroprotective properties [22], anti-proliferation properties [23], depicts antihyperuricemic activity [24] and anti-tumour properties [25]. Additionally, UA is currently available as a dietary supplement with a recommended daily intake between 150 and 300 mg/day. The average weight of an adult is 62 kg, which means the daily intake is between 2.4 mg/kg/day and 4.8 mg/kg/day.

Despite the interest in UA, a proper long-term toxicity study is missing. Hence this study was proposed. This study investigated the long-term effects of UA on overall health, organ function, blood chemistry, behaviour and motor skills. It is the first study that has researched the subchronic toxicity of UA under de OECD guidelines and good laboratory practice regulations. Furthermore, the dose levels chosen for this study cover the currently recommended daily intake of UA dietary supplements. All parameters that could bring up any toxic effects according to the book written by S.C. Gad in 2014 were tested.

The results showed that there was no significant difference between the test group and the different doses of the test groups (100 mg/kg/day, 300 mg/kg/day, 1000 mg/kg/day) for almost all of the (sub)parameters. All experimental groups had a decreased food intake for one timeframe, but it was back to normal at the next timeframe. Since it did not affect the body weight, and no abnormal behaviour was recorded, it seems unlikely that this incident was related to the administration of UA.

Moreover, the control group had a higher basic movement average than the experimental groups in both males and females. However, for the medium and low dose group, the difference was found because of isolated incidences, and there were no clinical observations notes, such as abnormal gait, increased/decreased activity or subdues behaviour that would suggest an effect on locomotor ability. The difference found between the high dose group and the control group in females was present before administration of UA and afterwards. Since the difference was already found before the 90-day trial, it is not surprising that the difference is still there afterwards so this difference is not related to the administration of UA. For males, there was only a difference between the high dose group and the control group in the average of basic movements, since this difference was not consistent throughout the other parameters (fine movements, X-ambulation and Y-ambulation), it was considered incidental to the administration of UA.

The platelet concentration was significantly higher compared to the control group for the mid-dose (300 mg/kg/day) and the high dose (1000 mg/kg/day) in males. Furthermore, the platelet concentration of the low dose was significantly lower than the control group and the mid-dose (300 mg/kg/day) was significantly higher than the low dose (100 mg/kg/day) in females. Although a difference was found in the platelet count, it does not necessarily mean that those animals were no longer healthy and/or UA had a toxic effect on these animals. A healthy Han-Wistar rat older than 17 weeks is considered to have a healthy platelet count if the values are between 574–1253 × 10^3^/μL. In addition to that, the healthy values for female Han-Wister rats older than 17 weeks are 599–1144 × 10^3^/μL [26]. Both males and females across all groups were well within these values, and the platelet concentration is not consistently higher or lower compared to the control group in both sexes, it seems unlikely that this change is related to the administration of UA.

Additionally, the medium-dose group of 300 mg/kg/day had a significantly higher bladder volume than the other experimental groups and the control group with an average of 5.03 ± 2.6 ml. However, there is no set range of a healthy bladder volume for Han-Wistar. The gross pathology and histopathology of the urinary bladder showed that there were no lesions or abnormalities found related to the administration of UA. Therefore, it seems unlikely that the increased bladder volume is a result of the administration of UA.

Furthermore, there was a difference found in the concentration of CK in both males and females where the mid and high dose groups had a significantly lower dose than the control group. CK is an intracellular enzyme and if present in plasma and can be used for quantitative analysis for heart conditions and muscle damage [27]. Values have been ranging from 100 U/L to 900 U/L in the control group [[28], [29], [30], [31], [32]], and the site of bleeding [33], measurement of plasma or blood [33] and analytical method [34,35] can cause variations in the measured value. None of the values were below 100 U/L in both males and females, and even though there is a decreased concentration of CK in the higher and/or medium-dose group, the concentrations are still considered healthy. Females had a lower concentration of CK than males, but the CK concentration in females are naturally lower than in males [36,37].

In addition to that, lesions were found during the gross pathology and histopathology on the thymus and lungs across both the control and the test groups in both males and females. This could indicate that UA did not influence the prevalence of lesions in both organs. Several studies have investigated the ‘natural’ prevalence of lesions in male and female Han-Wistar rats [38]. These studies showed that lesions in the thyroid and lung are not uncommon in healthy rats and therefore support our data.

## 7 Conclusion

Daily oral gavage administration of UA to male and female rats for 13 consecutive weeks was well tolerated and produced no effects on body weight, food consumption, clinical condition (including neurotoxicity assessment), haematology, coagulation, blood chemistry or macroscopic and microscopic pathology at dose levels up to and including 1000 mg/kg/day of UA compared to the vehicle control. The No Observed Effect Level (NOEL) for this study was greater than the dose of 1000 mg/kg/day.

## Competing interests

No declaration of competing and conflicting interests.

## Funding

This research was supported by iRiccorgpharm Health Pty Ltd. They had no influence in the results and statistical analysis.

## Authors’ contributions

The study was designed by Zhiyong He and Zhi-Cheng Xiao. Lotte Geerlofs wrote the manuscript on consultation with Zhiyong He and made revisions when necessary. Lotte and Sa Xiao analysed the data. All authors have read and approved the manuscript.

## CRediT authorship contribution statement

**Lotte Geerlofs:** Data curation, Writing - original draft, Writing - review & editing. **Zhiyong He:** Conceptualization, Methodology, Validation, Investigation, Resources, Writing - review & editing, Supervision, Project administration. **Sa Xiao:** Data curation, Funding acquisition. **Zhi-Cheng Xiao:** Conceptualization, Methodology, Resources, Supervision, Project administration, Funding acquisition.
