# Supplementary material for: Repeated dose (90 days) oral toxicity study of ursolic acid in Han-Wistar rats
Source: Toxicol Rep. 2020 May 8;7:610–23. doi: 10.1016/j.toxrep.2020.04.005 (PMC7229404; doi:10.1016/j.toxrep.2020.04.005)
Supplement: Supplementary file 1 [file mmc1.pdf]

Table A: Summary of the histopathology findings in alphabetic order; effect of UA on tissues A-E. N.V.L = no visible lesions. One animal showed infiltration of mononuclear cell in the epididymis in the high dose group in males.

		Male				Female			
		Group 1, Control	Group 2, 100 mg/kg/day	Group 3, 300 mg/kg/day	Group 4, 1000 mg/kg/day	Group 1, Control	Group 2, 100 mg/kg/day	Group 3, 300 mg/kg/day	Group 4, 1000 mg/kg/day
	**Number of animals**	10	10	10	10	10	10	10	10
**Artery, aorta**	Examined	10	0	0	10	10	0	0	10
	N.V.L	10	-	-	10	10	-	-	10
**Bone, femur**	Examined	10	0	0	10	10	0	0	10
	N.V.L	10	-	-	10	10	-	-	10
**Bone, sternum**	Examined	10	0	0	10	10	0	0	10
	N.V.L	10	-	-	10	10	-	-	10
**Bone marrow, femur**	Examined	10	0	0	10	10	0	0	10
	N.V.L	10	-	-	10	10	-	-	10
**Bone marrow, sternum**	Examined	10	0	0	10	10	0	0	10
	N.V.L	10	-	-	10	10	-	-	10
**Bone marrow, smear**	Examined	10	0	0	10	10	0	0	10
	N.V.L	10	-	-	10	10	-	-	10
**Brain**	Examined	10	0	0	10	10	0	0	10
	N.V.L	10	-	-	10	10	-	-	10
**Cervix**	Examined	-	-	-	-	10	0	0	10
	N.V.L	-	-	-	-	10	-	-	10
**Epididymis**	Examined	10	0	0	10	-	-	-	-
	N.V.L	10	-	-	9	-	-	-	-
	Infiltration, mononuclear cell minimal	0	-	-	1	-	-	-	-
**Esophagus**	Examined	10	0	0	10	10	0	0	10
	N.V.L	10	-	-	10	10	-	-	10
**Eye**	Examined	10	0	0	10	10	0	0	10
	N.V.L	10	-	-	10	10	-	-	10

Table B: Summary of the histopathology findings in alphabetic order; effect of UA on the galt and glands. N.V.L = no visible lesions. One male showed minimal infiltration of mononuclear cells in the harderian gland in the high dose group.

		Male				Female			
		Group 1, Control	Group 2, 100 mg/kg/day	Group 3, 300 mg/kg/day	Group 4, 1000 mg/kg/day	Group 1, Control	Group 2, 100 mg/kg/day	Group 3, 300 mg/kg/day	Group 4, 1000 mg/kg/day
	**Number of animals**	10	10	10	10	10	10	10	10
**Galt**	Examined	10	0	0	10	10	0	0	10
	N.V.L	10	-	-	10	10	-	-	10
**Gland, adrenal**	Examined	10	0	0	10	10	0	0	10
	N.V.L	10	-	-	10	10	-	-	10
**Gland, harderian**	Examined	10	0	0	10	10	0	0	10
	N.V.L	9	-	-	9	7	-	-	9
	Infiltration mononuclear cell minimal	1	-	-	1	0	-	-	1
	Mild	0	-	-	0	3	-	-	0
**Gland, lacrimal**	Examined	10	0	0	10	10	10	10	10
	N.V.L	10	-	-	0	10	10	10	9
	Mild atrophy	0	-	-	0	0	0	0	1
	Minimal infiltration mononuclear cell	0	-	-	1	0	0	0	0
**Gland, mammary**	Examined	9	0	0	10	10	0	0	10
	N.V.L	9	-	-	10	10	-	-	10
	Not present in section	1	0	0	0	-	-	-	-
**Gland, parathyroid**	Examined	10	0	0	8	9	0	0	6
	N.V.L	10	0	0	8	9	-	-	6
	Not present in section	0	-	-	2	1	0	0	4

Table C: Summary of the histopathology findings in alphabetic order; effect of UA on tissues G-K. N.V.L = no visible lesions. One male (control group) and one female (high dose group) showed minimal mononuclear cell infiltration in the kidney.

		Male				Female			
		**Group 1, Control**	**Group 2, 100 mg/kg/day**	**Group 3, 300 mg/kg/day**	**Group 4, 1000 mg/kg/day**	**Group 1, Control**	**Group 2, 100 mg/kg/day**	**Group 3, 300 mg/kg/day**	**Group 4, 1000 mg/kg/day**
	**Number of animals**	10	10	10	10	10	10	10	10
**Gland, pituitary**	Examined	10	0	0	10	9	0	0	10
	N.V.L	10	-	-	10	9	-	-	10
	Not present in second	-	-	-	-	1	0	0	0
**Gland, prostate**	Examined	10	0	0	10	-	-	-	-
	N.V.L	9	-	-	8	-	-	-	-
	Infiltration mononuclear minimal	1	-	-	2	-	-	-	-
**Gland, salivary, mandibular**	Examined	10	0	0	10	10	0	0	10
	N.V.L	10	-	-	10	10	-	-	10
**Gland, seminal vesicle**	Examined	10	0	0	10	-	-	-	-
	N.V.L	10	-	-	10	-	-	-	-
**Gland, thyroid**	Examined	10	0	0	10	0	0	10	10
	N.V.L	10	-	-	10	-	-	10	10
**Heart**	Examined	10	0	0	10	0	0	10	10
	N.V.L	10	-	-	10	-	-	10	10
	Minimal infiltration mononuclear cell	0	-	-	0			0	
**Kidney**	Examined	10	0	0	10	10	10	10	10
	N.V.L	9	-	-	10	10	0	0	9
	Degeneration; tubular	1	-	-	0	0	-	-	0
	Infiltration mononuclear cell minimal	1	-	-	0	0	-	-	1

Table D: Summary of the histopathology findings in alphabetic order; effect of UA on large intestines, liver, lung and mandibular lymph node. N.V.L = no visible lesions. Both males and females had lesions in/on the lungs across both the test groups and the control groups.

		Male				Female			
		Group 1, Control	Group 2, 100 mg/kg/day	Group 3, 300 mg/kg/day	Group 4, 1000 mg/kg/day	Group 1, Control	Group 2, 100 mg/kg/day	Group 3, 300 mg/kg/day	Group 4, 1000 mg/kg/day
	**Number of animals**	10	10	10	10	10	10	10	10
**Large intestine, cecum**	Examined	10	0	0	10	10	0	0	10
	N.V.L	10	-	-	10	10	-	-	10
**Large intestine, colon**	Examined	10	0	0	10	10	0	0	10
	N.V.L	10	-	-	10	10	-	-	10
**Large intestine, rectum**	Examined	10	0	0	10	10	0	0	10
	N.V.L	10	-	-	10	10	-	-	10
**Liver**	Examined	10	0	3	10	10	0	0	10
	N.V.L	9	-	3	8	4	-	-	5
	Minimal necrosis	0	-	0	0	1	-	-	0
	Minimal infiltration mononuclear cell	1	-	0	2	5	-	-	5
**Lung**	Examined	10	5	5	10	10	1	1	10
	N.V.L	5	2	3	7	9	1	0	10
	Minimal congestion	4	3	2	3	1	0	0	0
	Mild haemorrhage	0	0	0	0	0	0	1	0
	Minimal accumulation	2	0	0	0	0	0	0	0
**Lymph node, mandibular**	Examined	10	0	0	10	10	0	0	10
	N.V.L	10	-	-	10	10	-	-	10

Table E: Summary of the histopathology findings in alphabetic order; effect of UA on tissues L-S. N.V.L = no visible lesions. One male showed minimal infiltration of mononuclear cells in skeletal muscle and pancreas in the high dose group.

		Male				Female			
		Group 1, Control	Group 2, 100 mg/kg/day	Group 3, 300 mg/kg/day	Group 4, 1000 mg/kg/day	Group 1, Control	Group 2, 100 mg/kg/day	Group 3, 300 mg/kg/day	Group 4, 1000 mg/kg/day
	**Number of animals**	10	10	10	10	10	10	10	10
**Lymph node, mesenteric**	Examined	10	0	0	10	10	0	0	10
	N.V.L	10	-	-	10	10	-	-	10
**Muscle, skeletal**	Examined	10	0	0	10	10	0	0	10
	N.V.L	10	-	-	9	10	-	-	10
	Infiltration, mononuclear cell, minimal	10	-	-	1	0	-	-	0
**Nerve, optic**	Examined	0	0	0	10	10	0	0	10
	N.V.L	9	-	-	10	10	-	-	10
	Not examined, not present	1	0	0	10	-	-	-	-
**Nerve, sciatic**	Examined	10	0	0	10	10	0	0	10
	N.V.L	10	-	-	10	10	-	-	10
**Ovary**	Examined	-	-	-	-	10	0	0	10
	N.V.L	-	-	-	-	10	-	-	10
**Oviduct**	Examined	-	-	-	-	10	0	0	10
	N.V.L	-	-	-	-	10	-	-	10
**Pancreas**	Examined	10	0	0	10	10	0	0	10
	N.V.L	10	-	-	9	10	-	-	10
	Minimal mononuclear cell infiltration	0	-	-	1	0	-	-	0
**Skin**	Examined	10	0	0	10	10	0	0	10
	N.V.L	10	-	-	10	10	-	-	10
**Small intestines, duodenum**	Examined	10	0	0	10	10	0	0	10
	N.V.L	10	-	-	10	10	-	-	10

Table G: Summary of the histopathology findings in alphabetic order; effect of UA on tissues T-V.N.V.L = no visible lesions. One male showed minimal infiltration of mononuclear cells in the urinary bladder in the high dose group. One female had minimal congestion of the thymus in the high dose group.

		**Male**				**Female**			
		**Group 1, Control**	**Group 2, 100 mg/kg/day**	**Group 3, 300 mg/kg/day**	**Group 4, 1000 mg/kg/day**	**Group 1, Control**	**Group 2, 100 mg/kg/day**	**Group 3, 300 mg/kg/day**	**Group 4, 1000 mg/kg/day**
	**Number of animals**	10	10	10	10	10	10	10	10
**Thymus**	Examined	10	3	3	10	10	0	2	10
	N.V.L	6	3	0	5	6	-	2	9
	Minimal congestion	4	0	2	4	4	-	0	1
	Mild congestion	0	0	0	1	0	-	0	0
	Minimal haemorrhage	0	0	1	0	0	-	0	0
**Tong**	Examined	10	0	0	10	10	0	0	10
	N.V.L	10	-	-	10	10	-	-	10
**Trachea**	Submitted	10	0	0	10	10	0	0	10
	N.V.L	10	-	-	10	10	-	-	10
**Ureter**	Submitted	10	0	0	10	10	0	0	9
	N.V.L	10	-	-	10	10	-	-	9
	Not present in section	-	-	-	-	0			1
**Urinary bladder**	Submitted	10	0	0	10	10	0	0	10
	N.V.L	10	-	-	10	10	-	-	10
	Minimal infiltration mononuclear cell	0	-	-	1	0	-	-	0
**Uterus**	Examined	-	-	-	-	10	0	0	10
	N.V.L	-	-	-	-	10	-	-	10
	Cyst					1	-	-	0
**Vagina**	Submitted	-	-	-	-	10	0	0	10
	N.V.L	-	-	-	-	10	-	-	10
